# Scotochromogenic [sko′′to-kro′mo-jǝn-ik]

**DOI:** 10.3201/eid3107.230974

**Published:** 2025-07

**Authors:** Christoffel Opperman, Rob Warren

**Affiliations:** National Health Laboratory Service, Green Point TB-Laboratory, Cape Town, South Africa (C. Opperman); University of Cape Town, Cape Town (C. Opperman); SAMRC Centre for Tuberculosis Research, Stellenbosch University, Cape Town (R. Warren)

**Keywords:** Scotochromogenic, bacteria, nontuberculous mycobacteria, tuberculosis and other mycobacteria, *Mycobacterium gordonea*, *Suggested citation for this article*: Opperman C, Warren R. Etymologia: scotochromogenic. Emerg Infect Dis. 2025 Jul [*date cited*]. https://doi.org/10.3201/eid3107.230974

The scotochromogenic pigmentation pattern is named after the Greek terms σκότος (*skotos*, darkness), χρῶμα (*khrôma*, color), and γενής (*genḗs*, offspring or kind). The term is used to describe bacteria that form pigmentation without exposure to light. Pigmentation in the dark has the potential evolutionary advantage to increase microbial fitness by acting as antimicrobial agents, antioxidants, or virulence factors.

In 1959, US bacteriologist Ernest Runyon (1903–1994) classified nontuberculous mycobacteria into 4 groups based on growth rates, pigmentation, and colony morphology. Group II nontuberculous mycobacteria are slow-growing scotochromogens with the unique ability to produce pigmentation without light exposure; thus, they are scotochromogenic. Common group II members include *Mycobacterium szulgai*, named after the microbiologist Teofil Szulga (Ludwik Hirszfeld institute, Wroclaw, Poland) in 1962; *M. scrofulaceum* (Greek *scrofa* [sow, female pig]) named by Prissick and Masson (McGill University, Montreal, QB, Canada) in 1956; and *M. gordonea* (Figure), documented in 1962 in honor of the US bacterial taxonomist Ruth Evelyn Gordon (1910–2003) by Bojalil, Cerbon, and Trujillo (National Autonomous University of Mexico, Mexico City, Mexico).

**Figure:**
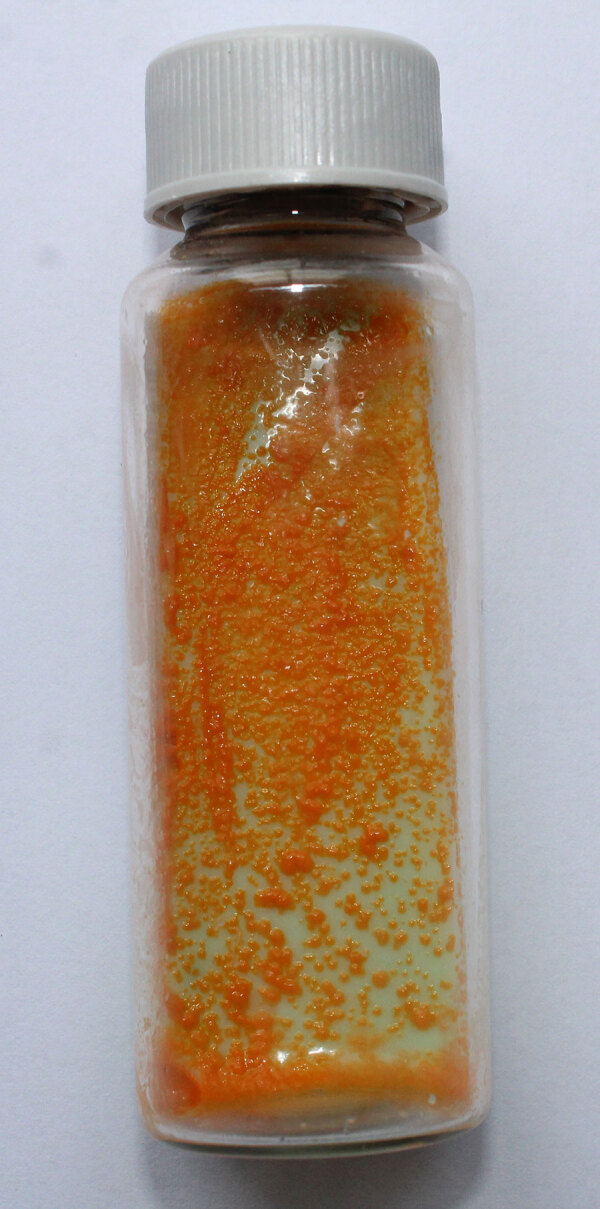
Slow-growing, scotochromogenic *Mycobacterium gordonea*, showing smooth, yellow-orange pigmented colonies. The sample was cultured aerobically on 8 mL of solid Löwenstein–Jensen media for 14 days at 37°C without exposure to light. This opportunistic nontuberculous mycobacterium was isolated repeatedly from the sputum of an immunocompromised, HIV-positive patient, who had an absolute CD4 count of 1. Sample processing and culture were performed at the National Health Laboratory Service, Green Point TB-laboratory, Cape Town, South Africa. Vial size is 28 mL. Photograph courtesy of the author.

Professor Gordon held a doctorate in bacteriology from Cornell University. In later years, the study of streptomycetes and aerobic spore-forming bacteria became her field of expertise, which led to her employment in the United States Department of Agriculture as a soil microbiologist. After World War II, she was a curator for the American Type Culture Collection (ATCC) Society beginning in 1947. She continued her work in recording bacterial collections as an ATCC visiting investigator even after her formal retirement in 1981. During the 1950s at Rutgers University, she pioneered the classifying, naming, and reliable descriptions of rapidly growing acid-fast bacteria. Because of her international reputation, she held various prestigious positions, including honorary president of the International Symposium on the Biology of Actinomycetes in Venezuela (1974) and Germany (1979). In addition, she received numerous accolades during her life, including the Alice Evans Award from the American Society of Microbiology (1992) and the J. Roger Porter Award from the US Federation for Culture Collections (1983).
